# Induced Pluripotent Stem Cells (iPSC) and Their Use in Disease Modeling

**DOI:** 10.7759/cureus.93999

**Published:** 2025-10-07

**Authors:** Alicja Dorota, Nicole Maryniak, Anna Mariankowska, Cezary Milczarek, Michal Dorota, Wojciech Zywiec, Karol Kozlowski, Illia Koval, Bartosz Czyzewski, Joanna Czyzewska

**Affiliations:** 1 Medical School, Zagłębiowskie Oncology Center, Dąbrowa Górnicza, POL; 2 Internal Medicine, Provincial Hospital of St. Luke, Tarnów, POL; 3 Internal Medicine, Medical University of Łódź, Łódź, POL; 4 Conservative Dentistry, Central Clinical Hospital of Medical University of Łódź, Łódź, POL; 5 Anesthesia and Critical Care, Provincial Integrated Hospital in Kielce, Kielce, POL; 6 Internal Medicine, Provincial Hospital in Poznań, Poznań, POL; 7 Internal Medicine, Central Clinical Hospital of Medical University of Łódź, Łódź, POL; 8 Rheumatology Clinic, Medical University of Łódź, Łódź, POL

**Keywords:** biomarkers, genetic editing, induced pluripotent stem cells (ipscs), in vitro experiments, regenerative therapy

## Abstract

This paper reviews methodologies for culturing induced pluripotent stem cells (iPSCs) and highlights their applications in disease modeling and regenerative medicine. iPSCs can be reprogrammed from multiple somatic cell sources, including keratinocytes, fibroblasts, peripheral blood mononuclear cells, and urinary epithelial cells. Their ability to differentiate into patient-specific cell types provides unique opportunities to model neurodegenerative, cardiovascular, metabolic, and autoimmune disorders in vitro. Pluripotency is typically induced by the overexpression of four canonical transcription factors-Oct4, Sox2, Klf4, and c-Myc. iPSC culture is technically demanding, as the cells display genomic and epigenetic instability and require tightly controlled microenvironmental conditions to maintain viability and pluripotency. Rigorous quality control, including PCR-based assays and genomic integrity analyses, is essential. Advances in iPSC technology have enabled personalized disease modeling, mechanistic studies of pathogenesis, drug screening, and the development of precision therapies. Despite their translational promise, iPSCs remain limited by issues of genomic instability, clinical safety, and the lack of standardized culture protocols.

## Introduction and background

Induced pluripotent stem cells (iPSCs) are somatic cells that have been genetically reprogrammed to acquire the defining properties of embryonic stem cells (ESCs). Reprogramming is achieved through the introduction of four transcription factors, Oct4, Sox2, Klf4, and c-Myc, which restore pluripotency and self-renewal capacity. iPSCs exhibit key stem cell characteristics, including ESC-like morphology, expression of pluripotency-associated transcriptional networks, specific surface antigen profiles, the ability to differentiate into derivatives of the three germ layers, and virtually unlimited proliferative potential. The development of iPSC technology by Shinya Yamanaka and colleagues in 2006 at Kyoto University represented a paradigm shift in regenerative medicine. By offering an ethically acceptable alternative to ESCs, iPSCs have provided an invaluable platform for basic biomedical research, disease modeling, pharmacological testing, and personalized cell-based therapies. In recognition of this groundbreaking discovery, Yamanaka was awarded the 2012 Nobel Prize in Physiology or Medicine [1]. We reviewed papers from oldest to newest, published in the most respected medical databases, and selected those that were most frequently cited and groundbreaking.

## Review

Somatic cell isolation

The initial step in generating iPSCs is the isolation of somatic cells from the donor. The choice of cell source is critical, as it directly influences the efficiency of reprogramming, the quality of the resulting iPSC lines, and their subsequent experimental or clinical applications [2]. Historically, dermal fibroblasts obtained from skin biopsies were the first cell type employed for iPSC generation. They remain a widely used starting material because, despite the invasiveness of biopsy collection, fibroblasts can be readily expanded, cryopreserved, and banked. Their high genomic stability makes them a reliable source for reprogramming [3]. An alternative, less invasive strategy is the isolation of peripheral blood mononuclear cells (PBMCs). These cells display comparable reprogramming efficiency to fibroblasts and are increasingly favored in translational studies due to the minimally invasive collection procedure [4,5]. More recently, urinary epithelial cells have emerged as an attractive source of somatic cells. They demonstrate robust reprogramming capacity while offering a completely non-invasive, reproducible, and easily repeatable method of sample acquisition, enabling the generation of multiple iPSC lines from the same donor within a short timeframe [6-8]. Keratinocytes derived from hair follicles have also been successfully utilized. Although this method yields fewer cells, the reprogramming efficiency is higher compared to fibroblasts. Other experimental sources include mesenchymal stromal cells isolated from dental pulp, synovial tissue, and hepatocytes; however, their application remains largely restricted to basic research contexts [9-11].

Pluripotency induction process

The generation of iPSCs is achieved by reprogramming somatic cells to a pluripotent state through the restoration of transcriptional and epigenetic programs characteristic of ESCs. This process reactivates the cells’ ability to differentiate into multiple lineages [12,13]. The introduction of four transcription factors, Oct4 (Pou5f1), Sox2, Klf4, and c-Myc, has been shown to be sufficient to revert fibroblasts to a pluripotent state in both murine and human cells [3]. Early approaches relied on retroviral and lentiviral vectors, which were highly efficient but carried the risk of transgene integration into the host genome, thereby increasing the likelihood of insertional mutagenesis and tumorigenesis. To mitigate these risks, integration-free methods have been developed, including episomal DNA vectors, synthetic mRNA, recombinant protein delivery, and Sendai virus-based systems. Although less efficient, these strategies significantly enhance biosafety [14,15]. Reprogramming involves two principal mechanisms: chromatin remodeling and DNA methylation resetting. Initially, the transcriptional program of the somatic cell is silenced, followed by activation of pluripotency-associated genes. Endogenous reactivation of the Oct4 promoter serves as the central stabilizing mechanism of the pluripotent state. The efficiency of this process, which typically requires several days to weeks, remains low (<0.1-several percent), depending on both technical factors (vector type, transfection method) and biological factors (donor age, cell type, epigenetic profile) [16,17]. Younger donor cells are generally reprogrammed with higher efficiency, likely due to reduced accumulation of epigenetic alterations [18-20]. A major challenge during reprogramming is the preservation of genomic stability. The forced expression of transcription factors can induce mutations and DNA damage, compromising cell function and differentiation capacity. Therefore, continuous genomic monitoring is essential throughout the reprogramming and culture process [21,22].

iPSC culture

Following successful reprogramming, the maintenance of iPSCs under in vitro culture conditions is essential to preserve their proliferative capacity and pluripotency. Suboptimal environments may trigger spontaneous differentiation or loss of stem cell properties, significantly limiting downstream applications [23-25]. Early iPSC culture protocols employed feeder layers composed of mitotically inactivated mouse embryonic fibroblasts, which secreted supportive factors. However, to enhance reproducibility and minimize xenogeneic contamination, feeder-free systems are increasingly used. These rely on extracellular matrix coatings such as Matrigel or recombinant human proteins, including laminin [26-28]. The culture medium typically consists of chemically defined formulations, such as mTeSR1 or E8, supplemented with essential growth factors (e.g., FGF2) and inhibitors of differentiation pathways (e.g., TGF-β/activin A). These media enable greater standardization and are considered more suitable for translational and clinical applications [29,30]. To sustain long-term viability, iPSCs require routine passaging, either mechanically or enzymatically (e.g., dispase, EDTA). Long-term cryopreservation is achieved using cryoprotectants such as 10% DMSO (dimethyl sulfoxide), allowing stable reconstitution of iPSC lines after thawing [31,32]. Despite advances in culture technology, challenges persist. iPSCs remain susceptible to genomic instability, particularly during extended passaging. Furthermore, small fluctuations in medium composition or environmental conditions may induce spontaneous differentiation, complicating process standardization [33,34]. Current efforts aim to establish xeno-free, chemically defined, and standardized culture conditions to support clinical translation [35].

Monitoring iPSC quality

Rigorous quality control is essential to verify the pluripotent state of iPSCs. Expression of canonical pluripotency markers, including Oct4 and Nanog, is typically assessed via PCR, immunocytochemistry, or flow cytometry. Functional pluripotency is confirmed by directed differentiation assays into all three germ layers (ectoderm, mesoderm, endoderm), generating representative cell types such as neurons, cardiomyocytes, and myocytes [36,37]. In addition, genomic integrity must be regularly evaluated, as reprogramming can introduce chromosomal abnormalities or epigenetic alterations. Such changes may compromise differentiation efficiency or predispose cells to malignant transformation [38,39].

Application of iPSCs in disease modeling

iPSCs have become an indispensable tool for modeling human diseases in vitro, enabling the generation of patient-specific cellular models that recapitulate disease pathophysiology. These models facilitate the study of molecular mechanisms, biomarker discovery, drug testing, and the development of personalized therapeutic strategies [40]. Furthermore, autologous iPSC-derived tissues reduce the risk of immune rejection in regenerative applications [41].

Neurodegenerative Diseases

iPSC-derived neuronal models have provided new insights into Alzheimer’s disease (AD), Parkinson’s disease (PD), and amyotrophic lateral sclerosis (ALS). Patient-specific neurons allow the analysis of pathogenic mechanisms and the evaluation of pharmacological interventions. For ALS, iPSCs have enabled the identification of disease biomarkers and therapeutic compounds. In AD, iPSC-derived neurons and glia reproduce hallmarks such as tau hyperphosphorylation and β-amyloid deposition, offering a platform for targeted therapeutic development [42-44]. PD models have recapitulated dopaminergic neuron degeneration in the substantia nigra and revealed the pathogenic role of α-synuclein aggregation, advancing the understanding of both sporadic and familial PD [45-47].

Cardiovascular Diseases

iPSCs differentiated into cardiomyocytes enable the study of arrhythmogenic disorders, heart failure, and myocardial injury. For example, models of congenital arrhythmias linked to KCNQ1 mutations provide a basis for precision cardiology [48-50]. In myocardial damage, iPSC-derived cardiomyocytes, fibroblasts, vascular smooth muscle cells, and endothelial cells have been explored for regenerative transplantation strategies, with promising improvements in cardiac function [1,51-53].

Metabolic Diseases

iPSCs represent a powerful platform for studying genetic and metabolic diseases, as they preserve the patient’s genotype in vitro [54]. In cystic fibrosis, iPSC-derived airway epithelial cells reproduce defective chloride transport and excessive mucus secretion caused by CFTR mutations, facilitating the evaluation of targeted drugs such as ivacaftor and lumacaftor [55-57]. In Duchenne muscular dystrophy (DMD), iPSC-derived myocytes allow mechanistic studies of muscle degeneration, and gene editing has restored dystrophin expression in vitro, highlighting therapeutic potential [58-60]. Wilson’s disease has also been modeled using iPSC-derived hepatocytes, which reproduce copper accumulation and oxidative stress, providing a platform for preclinical drug testing [61-63].

Autoimmune Diseases

Modeling autoimmune disorders has historically been challenging due to the complexity of the immune microenvironment. iPSCs provide novel opportunities to overcome these barriers [64]. In systemic lupus erythematosus (SLE), iPSC-derived B and T lymphocytes exhibit dysregulated signaling and enhanced autoantibody production, recapitulating disease-specific immune dysfunction [65]. In rheumatoid arthritis, iPSC-derived fibroblast-like synoviocytes reproduce the proinflammatory phenotype driving joint destruction, enabling the screening of targeted inhibitors [66,67]. For type 1 diabetes mellitus, iPSCs have been differentiated into insulin-producing β-like cells, which, when co-cultured with patient-derived T cells, reproduce autoimmune destruction of pancreatic islets [68,69]. In multiple sclerosis (MS), iPSC-derived oligodendrocytes replicate demyelination and remyelination processes, facilitating investigations into neuroprotective and immunomodulatory interventions [70,71].

**Table 1:** The use of iPSCs in the above diseases. DMD: Duchenne muscular dystrophy; SLE: systemic lupus erythematosus; MS: multiple sclerosis

Disease		Use of iPSCs
Neurodegenerative disease	Alzheimer's disease	Studying the effect of mutations on increased tau protein phosphorylation and beta-amyloid accumulation to find effective treatments [4,44,45]
	Parkinson's disease	Studying the accumulation of alpha-synuclein in neurons to develop new therapies [3,46,47]
Heart Disease	Arrhythmias	The effect of mutations in the KCNQ1 gene on the development of congenital cardiac arrhythmias [48-50]
	Myocardial damage	Replacement transplantation of the resulting cardiomyocytes, fibroblasts, vascular smooth muscle, or endothelial cells [1,51-53]
Metabolic diseases	Cystic fibrosis	Reproduction of chloride transport defects and excessive mucus production caused by CFTR mutations and testing of new drugs, i.e., ivacaftor and lumacaftor [55-57]
	DMD	The dystrophin gene mutation and the mechanisms of muscle fiber degeneration were investigated, allowing for the development of new therapies [58-60]
	Wilson's disease	The ATP7B gene mutations were investigated, allowing for the testing of new drugs and the assessment of their effectiveness [61-63]
Autoimmune diseases	SLE	Investigation of defective B and T lymphocyte regulation and interactions with antigen-presenting cells [65]
	Rheumatoid arthritis	Obtaining fibroblast-like synoviocytes, which enabled the study of new signaling pathway inhibitors [66,67]
	Type I diabetes	Creation of functional insulin-producing cells and replication of the autoimmune attack on pancreatic islets in vitro [68,69]
	MS	Creation of oligodendrocytes and replication of remyelination and demyelination in vitro [70,71]

Challenges and limitations of iPSC technology

Despite their transformative potential, iPSCs face significant technical and translational challenges that limit their widespread application in disease modeling and regenerative medicine. A major obstacle remains the low efficiency of somatic cell reprogramming, which is influenced by donor age, cell type, and the reprogramming strategy employed [72]. Furthermore, heterogeneity among iPSC lines complicates standardization and reduces reproducibility across laboratories. Genomic alterations, including chromosomal deletions, duplications, and translocations, frequently arise during long-term culture and can compromise differentiation capacity or promote neoplastic transformation [72]. Another unresolved issue is the so-called epigenetic memory of donor cells, wherein residual DNA methylation and histone modification patterns restrict full reprogramming and impair lineage-specific differentiation [73]. The risk of tumorigenesis remains one of the most serious barriers to clinical translation. Undifferentiated iPSCs within transplant populations can give rise to teratomas, while earlier viral-based reprogramming methods posed additional risks by integrating exogenous sequences into the host genome [38]. Although non-integrating systems have improved safety, the risk has not been fully eliminated. Another limitation is the incomplete maturation of iPSC-derived cells. Many exhibit a fetal-like phenotype, which restricts their utility for modeling late-onset and adult diseases. The lack of universally accepted protocols for differentiation and the absence of global quality control standards further hinder clinical progress [74]. Moreover, in vitro iPSC-derived models often fail to recapitulate the complex in vivo microenvironment, including interactions with the immune and endocrine systems. Consequently, while these models offer powerful tools to study disease mechanisms, they often require simplification that may not fully capture disease pathophysiology.

## Conclusions

iPSCs represent one of the most significant innovations in experimental and translational medicine. Their ability to differentiate into nearly any cell type, combined with the possibility of generating patient-specific models, has provided unprecedented opportunities for disease modeling, mechanistic studies, and therapeutic development. iPSC-based models have already contributed to breakthroughs in understanding neurodegenerative, cardiovascular, metabolic, and autoimmune diseases. Nonetheless, iPSCs are not without limitations. Issues of genomic and epigenetic instability, risk of tumorigenesis, and difficulties in achieving complete cellular maturation remain major challenges. Standardization of culture conditions and establishment of rigorous quality control criteria are essential for their safe clinical application. Future directions include the integration of iPSC technology with genome editing tools, organoid systems, and microfluidic platforms, which may allow more physiologically relevant modeling of human tissues and accelerate the development of personalized therapies. Although numerous challenges remain, the continued refinement of iPSC methodologies holds great promise for advancing precision medicine and regenerative therapeutics.

## References

[1] Kolanowski T, Kurpisz M (2010). Induced pluripotential stem cells — perspectives of clinical application in cardiovascular diseases [article in Polish]. Kardiol Pol.

[2] Park J, Kim J, Shin B, Sch Ler HR, Kim J, Kim KP (2024). Inducing pluripotency in somatic cells: historical perspective and recent advances. Int J Stem Cells.

[3] Takahashi K, Yamanaka S (2006). Induction of pluripotent stem cells from mouse embryonic and adult fibroblast cultures by defined factors. Cell.

[4] Loh YH, Hartung O, Li H (2010). Reprogramming of T cells from human peripheral blood. Cell Stem Cell.

[5] El Hokayem J, Cukier HN, Dykxhoorn DM (2016). Blood derived induced pluripotent stem cells (iPSCs): benefits, challenges and the road ahead. J Alzheimers Dis Parkinsonism.

[6] Shi T, Cheung M (2021). Urine-derived induced pluripotent/neural stem cells for modeling neurological diseases. Cell Biosci.

[7] Steinle H, Weber M, Behring A (2019). Reprogramming of urine-derived renal epithelial cells into iPSCs using srRNA and consecutive differentiation into beating cardiomyocytes. Mol Ther Nucleic Acids.

[8] Zhou T, Benda C, Dunzinger S (2012). Generation of human induced pluripotent stem cells from urine samples. Nat Protoc.

[9] Raab S, Klingenstein M, Liebau S, Linta L (2014). A comparative view on human somatic cell sources for iPSC generation. Stem Cells Int.

[10] Yan X, Qin H, Qu C, Tuan RS, Shi S, Huang GT (2010). iPS cells reprogrammed from human mesenchymal-like stem/progenitor cells of dental tissue origin. Stem Cells Dev.

[11] Sauer V, Roy-Chowdhury N, Guha C, Roy-Chowdhury J (2014). Induced pluripotent stem cells as a source of hepatocytes. Curr Pathobiol Rep.

[12] van den Hurk M, Kenis G, Bardy C, van den Hove DL, Gage FH, Steinbusch HW, Rutten BP (2016). Transcriptional and epigenetic mechanisms of cellular reprogramming to induced pluripotency. Epigenomics.

[13] Brix J, Zhou Y, Luo Y (2015). The epigenetic reprogramming roadmap in generation of iPSCs from somatic cells. J Genet Genomics.

[14] Zhong C, Liu M, Pan X, Zhu H (2022). Tumorigenicity risk of iPSCs in vivo: nip it in the bud. Precis Clin Med.

[15] Manzini S, Viiri LE, Marttila S, Aalto-Setälä K (2015). A comparative view on easy to deploy non-integrating methods for patient-specific iPSC production. Stem Cell Rev Rep.

[16] Brambrink T, Foreman R, Welstead GG, Lengner CJ, Wernig M, Suh H, Jaenisch R (2008). Sequential expression of pluripotency markers during direct reprogramming of mouse somatic cells. Cell Stem Cell.

[17] Tian Z, Guo F, Biswas S, Deng W (2016). Rationale and methodology of reprogramming for generation of induced pluripotent stem cells and induced neural progenitor cells. Int J Mol Sci.

[18] Trokovic R, Weltner J, Noisa P, Raivio T, Otonkoski T (2015). Combined negative effect of donor age and time in culture on the reprogramming efficiency into induced pluripotent stem cells. Stem Cell Res.

[19] Ravaioli F, Bacalini MG, Franceschi C, Garagnani P (2018). Age-related epigenetic derangement upon reprogramming and differentiation of cells from the elderly. Genes (Basel).

[20] Mackey LC, Annab LA, Yang J (2018). Epigenetic enzymes, age, and ancestry regulate the efficiency of human iPSC reprogramming. Stem Cells.

[21] Ruiz S, Lopez-Contreras AJ, Gabut M (2015). Limiting replication stress during somatic cell reprogramming reduces genomic instability in induced pluripotent stem cells. Nat Commun.

[22] Rossi A, Lickfett S, Martins S, Prigione A (2022). A call for consensus guidelines on monitoring the integrity of nuclear and mitochondrial genomes in human pluripotent stem cells. Stem Cell Reports.

[23] Eidhof I, Ulfenborg B, Kele M, Shahsavani M, Winn D, Uhlén P, Falk A (2025). Defined culture conditions robustly maintain human stem cell pluripotency, highlighting a role for Ca(2+) signaling. Commun Biol.

[24] Matsuo-Takasaki M, Kambayashi S, Hemmi Y (2024). Complete suspension culture of human induced pluripotent stem cells supplemented with suppressors of spontaneous differentiation. Elife.

[25] Yamamoto T, Arita M, Kuroda H, Suzuki T, Kawamata S (2022). Improving the differentiation potential of pluripotent stem cells by optimizing culture conditions. Sci Rep.

[26] Jiang G, Wan X, Wang M, Zhou J, Pan J, Wang B (2016). A reliable and economical method for gaining mouse embryonic fibroblasts capable of preparing feeder layers. Cytotechnology.

[27] Jozefczuk J, Drews K, Adjaye J (2012). Preparation of mouse embryonic fibroblast cells suitable for culturing human embryonic and induced pluripotent stem cells. J Vis Exp.

[28] Crocco MC, Fratnz N, Bos-Mikich A (2013). Substrates and supplements for hESCs: a critical review. J Assist Reprod Genet.

[29] Chen G, Gulbranson DR, Hou Z (2011). Chemically defined conditions for human iPSC derivation and culture. Nat Methods.

[30] Chen KG, Mallon BS, McKay RD, Robey PG (2014). Human pluripotent stem cell culture: considerations for maintenance, expansion, and therapeutics. Cell Stem Cell.

[31] Cruvinel E, Ogusuku I, Cerioni R (2020). Long-term single-cell passaging of human iPSC fully supports pluripotency and high-efficient trilineage differentiation capacity. SAGE Open Med.

[32] Uhrig M, Ezquer F, Ezquer M (2022). Improving cell recovery: Freezing and thawing optimization of induced pluripotent stem cells. Cells.

[33] Yoshihara M, Oguchi A, Murakawa Y (2019). Genomic instability of iPSCs and challenges in their clinical applications. Adv Exp Med Biol.

[34] Peterson SE, Loring JF (2014). Genomic instability in pluripotent stem cells: implications for clinical applications. J Biol Chem.

[35] Lu HF, Chai C, Lim TC (2014). A defined xeno-free and feeder-free culture system for the derivation, expansion and direct differentiation of transgene-free patient-specific induced pluripotent stem cells. Biomaterials.

[36] Akopian V, Andrews PW, Beil S (2010). Comparison of defined culture systems for feeder cell free propagation of human embryonic stem cells. In Vitro Cell Dev Biol Anim.

[37] Hua Y, Yoshimochi K, Li J (2022). Development and evaluation of a novel xeno-free culture medium for human-induced pluripotent stem cells. Stem Cell Res Ther.

[38] Poetsch MS, Strano A, Guan K (2022). Human induced pluripotent stem cells: from cell origin, genomic stability, and epigenetic memory to translational medicine. Stem Cells.

[39] Liang G, Zhang Y (2013). Genetic and epigenetic variations in iPSCs: potential causes and implications for application. Cell Stem Cell.

[40] Paik DT, Chandy M, Wu JC (2020). Patient and disease-specific induced pluripotent stem cells for discovery of personalized cardiovascular drugs and therapeutics. Pharmacol Rev.

[41] Medvedev SP, Shevchenko AI, Zakian SM (2010). Induced pluripotent stem cells: problems and advantages when applying them in regenerative medicine. Acta Naturae.

[42] Penney J, Ralvenius WT, Tsai LH (2020). Modeling Alzheimer's disease with iPSC-derived brain cells. Mol Psychiatry.

[43] Guo X, Wang X, Wang J, Ma M, Ren Q (2025). Current development of iPSC-based modeling in neurodegenerative diseases. Int J Mol Sci.

[44] Cao L, Tan L, Jiang T, Zhu XC, Yu JT (2015). Induced pluripotent stem cells for disease modeling and drug discovery in neurodegenerative diseases. Mol Neurobiol.

[45] Avazzadeh S, Baena JM, Keighron C, Feller-Sanchez Y, Quinlan LR (2021). Modelling Parkinson’s disease: iPSCs towards better understanding of human pathology. Brain Sci.

[46] Singh Dolt K, Hammachi F, Kunath T (2017). Modeling Parkinson's disease with induced pluripotent stem cells harboring α-synuclein mutations. Brain Pathol.

[47] Byers B, Lee HL, Reijo Pera R (2012). Modeling Parkinson's disease using induced pluripotent stem cells. Curr Neurol Neurosci Rep.

[48] Vo QD, Nakamura K, Saito Y, Akagi S, Miyoshi T, Yuasa S (2025). Induced pluripotent stem cells in cardiomyopathy: advancing disease modeling, therapeutic development, and regenerative therapy. Int J Mol Sci.

[49] Ren L, Jahng JW, Belbachir N, Cook Z, Rivero GC, Perez MV, Wu JC (2024). Generation of induced pluripotent stem cell lines from patients with LQT1 caused by heterozygous mutations in the KCNQ1 gene. Stem Cell Res.

[50] Davis J, Chouman A, Creech J (2021). In vitro model of ischemic heart failure using human induced pluripotent stem cell-derived cardiomyocytes. JCI Insight.

[51] Lalit PA, Hei DJ, Raval AN, Kamp TJ (2014). Induced pluripotent stem cells for post-myocardial infarction repair: remarkable opportunities and challenges. Circ Res.

[52] Ayoubi S, Sheikh SP, Eskildsen TV (2017). Human induced pluripotent stem cell-derived vascular smooth muscle cells: differentiation and therapeutic potential. Cardiovasc Res.

[53] Xiao Y, Chen Y, Shao C, Wang Y, Hu S, Lei W (2022). Strategies to improve the therapeutic effect of pluripotent stem cell-derived cardiomyocytes on myocardial infarction. Front Bioeng Biotechnol.

[54] Ramachandra CJ, Chua J, Cong S, Kp MM, Shim W, Wu JC, Hausenloy DJ (2021). Human-induced pluripotent stem cells for modelling metabolic perturbations and impaired bioenergetics underlying cardiomyopathies. Cardiovasc Res.

[55] Firth AL, Menon T, Parker GS (2015). Functional gene correction for cystic fibrosis in lung epithelial cells generated from patient iPSCs. Cell Rep.

[56] Wong AP, Bear CE, Chin S (2012). Directed differentiation of human pluripotent stem cells into mature airway epithelia expressing functional CFTR protein. Nat Biotechnol.

[57] Harutyunyan M, Huang Y, Mun KS, Yang F, Arora K, Naren AP (2018). Personalized medicine in CF: from modulator development to therapy for cystic fibrosis patients with rare CFTR mutations. Am J Physiol Lung Cell Mol Physiol.

[58] Jin Y, Shen Y, Su X, Weintraub N, Tang Y (2019). CRISPR/Cas9 technology in restoring dystrophin expression in iPSC-derived muscle progenitors. J Vis Exp.

[59] Jin Y, Shen Y, Su X, Weintraub NL, Tang Y (2020). Effective restoration of dystrophin expression in iPSC (Mdx)-derived muscle progenitor cells using the CRISPR/Cas9 system and homology-directed repair technology. Comput Struct Biotechnol J.

[60] Cai WF, Huang W, Wang L (2016). Induced pluripotent stem cells derived muscle progenitors effectively mitigate muscular dystrophy through restoring the dystrophin distribution. J Stem Cell Res Ther.

[61] Petters J, Völkner C, Krohn S (2020). Generation of two induced pluripotent stem cell lines from a female adult homozygous for the Wilson disease associated ATP7B variant p.H1069Q (AKOSi008-A) and a healthy control (AKOSi009-A). Stem Cell Res.

[62] Song D, Takahashi G, Zheng YW (2022). Retinoids rescue ceruloplasmin secretion and alleviate oxidative stress in Wilson's disease-specific hepatocytes. Hum Mol Genet.

[63] Zhang S, Chen S, Li W (2011). Rescue of ATP7B function in hepatocyte-like cells from Wilson's disease induced pluripotent stem cells using gene therapy or the chaperone drug curcumin. Hum Mol Genet.

[64] Hew M, O'Connor K, Edel MJ, Lucas M (2015). The possible future roles for iPSC-derived therapy for autoimmune diseases. J Clin Med.

[65] Li W, Liu D, Zheng F (2021). Generation of systemic lupus erythematosus patient-derived induced pluripotent stem cells from blood. Stem Cells Dev.

[66] Bottini N, Firestein GS (2013). Duality of fibroblast-like synoviocytes in RA: passive responders and imprinted aggressors. Nat Rev Rheumatol.

[67] Fang Z, Lv J, Wang J (2020). C-reactive protein promotes the activation of fibroblast-like synoviocytes from patients with rheumatoid arthritis. Front Immunol.

[68] Kim MJ, Lee EY, You YH, Yang HK, Yoon KH, Kim JW (2020). Generation of iPSC-derived insulin-producing cells from patients with type 1 and type 2 diabetes compared with healthy control. Stem Cell Res.

[69] Armitage LH, Stimpson SE, Santostefano KE (2021). Use of induced pluripotent stem cells to build isogenic systems and investigate type 1 diabetes. Front Endocrinol (Lausanne).

[70] Morales Pantoja IE, Smith MD, Rajbhandari L (2020). iPSCs from people with MS can differentiate into oligodendrocytes in a homeostatic but not an inflammatory milieu. PLoS One.

[71] Czepiel M, Boddeke E, Copray S (2015). Human oligodendrocytes in remyelination research. Glia.

[72] Moy AB, Kamath A, Ternes S, Kamath J (2023). The challenges to advancing induced pluripotent stem cell-dependent cell replacement therapy. Med Res Arch.

[73] Papp B, Plath K (2013). Epigenetics of reprogramming to induced pluripotency. Cell.

[74] Attwood SW, Edel MJ (2019). iPS-cell technology and the problem of genetic instability-can it ever be safe for clinical use?. J Clin Med.

